# The Intraflagellar Transport Protein IFT27 promotes BBSome exit from cilia through the GTPase ARL6/BBS3

**DOI:** 10.1186/2046-2530-4-S1-O18

**Published:** 2015-07-13

**Authors:** G Liew, F Ye, A Nager, J Murphy, J Lee, M Aguiar, D Breslow, S Gygi, M Nachury

**Affiliations:** 1 Department of Biochemistry, Stanford University School of Medicine, Stanford, CA, USA; 2 Department of Molecular and Cellular Physiology, Stanford University School of Medicine, Stanford, CA, USA; 3 Department of Cell Biology, Harvard Medical School, Cambridge, MA, USA

## Objective

To dissect the regulation of ciliary trafficking by small GTPases.

## Methods

Proteomics, Enzymology, Immunocytochemistry, Live-cell photokinetics.

## Results

Upon disengagement from the IFT-B complex, the IFT27/RabL4 subunit directly and specifically recognizes nucleotide-empty ARL6.

IFT27 stabilizes nucleotide-empty ARL6 against aggregation, supporting a role for IFT27 in promoting nucleotide exchange on ARL6.

Immunocytochemistry on IFT27-depleted cells reveals hyperaccumulation of ARL6 and BBSome in cilia.

Direct measurements of ciliary entry and exit rates show that IFT27 promotes BBSome exit out of cilia with no influence on entry, thus placing the site of IFT27 action within cilia.

While the BBSome is normally associated with IFT trains inside cilia, most of the BBSome is dissociated from IFT trains in IFT-depleted cells.

A putative BBSome cargo, the Hedgehog signaling intermediate GPR161, accumulates inside cilia of IFT27 and ARL6 knockout cells.

## Conclusions

Our data suggest that upon disassembly of IFT/BBSome trains at the tip, the IFT27 subunit transiently detaches from the IFT complex to participate in GTP loading onto ARL6, which then triggers formation of a retrograde BBSome coat for trafficking of the BBSome and its associated cargoes out of cilia. In other words, the disassembly of an anterograde IFT/BBSome train produces the trigger for assembly of the future retrograde IFT/BBSome train.

